# Acute and Fatal Isoniazid-Induced Hepatotoxicity: A Case Report and Review of the Literature

**DOI:** 10.1155/2016/3617408

**Published:** 2016-08-25

**Authors:** Wissam K. Kabbara, Aline T. Sarkis, Paola G. Saroufim

**Affiliations:** ^1^Department of Pharmacy Practice, School of Pharmacy, Lebanese American University (LAU), P.O. Box 36/F-37, Byblos, Lebanon; ^2^School of Pharmacy, Lebanese American University (LAU), Byblos, Lebanon

## Abstract

This paper describes a case of an acute and fatal isoniazid-induced hepatotoxicity and provides a review of the literature. A 65-year-old female diagnosed with latent* Mycobacterium tuberculosis* infection was receiving oral isoniazid 300 mg daily. She was admitted to the hospital for epigastric and right sided flank pain of one-week duration. Laboratory results and imaging confirmed hepatitis. After ruling out all other possible causes, she was diagnosed with isoniazid-induced acute hepatitis (probable association by the Naranjo scale). After discharge, the patient was readmitted and suffered from severe coagulopathy, metabolic acidosis, acute kidney injury, hepatic encephalopathy, and cardiorespiratory arrest necessitating two rounds of cardiopulmonary resuscitation. Despite maximal hemodynamic support, the patient did not survive. A review of the literature, from several European countries and the United States of America, revealed a low incidence of mortality due to isoniazid-induced hepatotoxicity when used as a single agent for latent* Mycobacterium tuberculosis* infection. As for the management, the first step consists of withdrawing isoniazid and rechallenge is usually discouraged. Few treatment modalities have been proposed; however there is no robust evidence to support any of them. Routine monitoring for hepatotoxicity in patients receiving isoniazid is warranted to prevent morbidity and mortality.

## 1. Introduction

Isoniazid (INH) is an antituberculosis agent that is commonly used for the treatment and prophylaxis of tuberculosis (TB). It inhibits bacterial cell wall synthesis, thus killing* Mycobacterium tuberculosis* organisms. Due to its significant potential in reducing morbidity, the use of INH in treating latent* Mycobacterium tuberculosis* infection (LTBI) is recommended as a first-line option since the 1965 American Thoracic Society guidelines especially in high risk individuals [1].

Since its introduction in 1952, several cases of INH-induced hepatotoxicity were reported [2–11]. Drug-induced liver injury guidelines caused by INH appears to be due to metabolic idiosyncratic reactions. INH is metabolized mostly by the liver, primarily by acetylation by N-acetyl transferase 2 (NAT-2) to acetyl-isoniazid. Acetyl-isoniazid is metabolized mainly to monoacetyl hydrazine (MAH) and to the nontoxic diacetyl hydrazine, as well as other minor metabolites. The influence of acetylation rate on INH hepatotoxicity is controversial; however, the involvement of INH's metabolites has been proposed: first by free radical generation from reactive metabolites of MAH and second by the covalent bond of acetyl hydrazine to liver macromolecules. Patients with enhanced cytochrome P450 2E1 activity carrying the homozygous cytochrome P450 2E1 c1/c1 host gene polymorphism showed an increased risk of developing hepatotoxicity particularly if known to be slow acetylators [12].

Multiple risk factors may contribute to INH-related hepatotoxicity. Some of them have conflicting evidence such as racial difference [13–15] and female gender [16, 17]. Other risk factors are well-confirmed including age [13, 18, 19], alcohol consumption [14, 15], and the concomitant administration of other hepatotoxic drugs such as acetaminophen [20], methotrexate [21], sulfasalazine [21], or carbamazepine [22].

Reports of severe and fatal hepatitis associated with INH emerged after the 1970 TB outbreak in Capitol Hill in Washington, DC, among workers who were receiving it for prophylaxis. Nineteen patients developed signs and symptoms of liver damage; and two of them died [23].

Accordingly, the guidelines published in 1971 were updated to include pretreatment screening and monitoring to reduce the risk of such complications [24]. In 1979, the USPHS trial showed a rate of death due to INH of 0.06% [15]. The IUAT study, a large eastern European clinical trial, estimated the rates of fatal INH hepatitis as 14 per 100,000 person-years [25]. Again, the 1983 guidelines were further reviewed to recommend routine clinical and laboratory monitoring for patients who are older than 35 years and for those with additional risk factors for hepatotoxicity [26]. Another report published in 1992 revealed rates of INH fatal hepatitis of around 0.02% which is lower than the incidence stated in the USPHS trial [17]. Salpeter evaluated articles published from 1966 to 1992 on INH use (with or without other antituberculosis drugs) in chemoprophylaxis. All patients were appropriately monitored according to the guidelines. Results showed 2 hepatotoxic deaths in 202,497 patients (an adjusted fatality rate of 0.003%). Both patients were taking INH monotherapy but had additional risk factors for hepatotoxicity [27]. In 1996, Millard et al. estimated rates of fatal INH hepatitis to be around 4.2 per 100,000 persons newly starting therapy and around 7 per 100,000 while completing therapy [28].

The most recent American Thoracic Society guidelines (2003) recommends INH 300 mg monotherapy daily for 9 months for human immunodeficiency virus (HIV) negative and positive individuals. Routine baseline and follow-up laboratory monitoring is only recommended for those who are HIV positive, have chronic liver disease, and are regular alcohol consumers or pregnant [29]. A recent case report (2015) was published presenting the case of a 53-year-old Japanese male diagnosed with INH-induced acute liver failure during a course of preventive therapy for LTBI [30]. Liver transplant was refused by the patient and his family leading to his death 4 months after admission.

Here, we present a probable case, as determined by the Naranjo adverse drug reaction probability scale score [31], of INH-induced fulminant hepatic failure in a 65-year-old woman who was receiving INH for LTBI.

## 2. Case Presentation

We present the case of a 65-year-old (weight, 80 Kg, height, 150 cm) Sri Lankan black woman, known to have LTBI treated with INH and dyslipidemia. She was diagnosed with LTBI based on a positive purified protein derivative test. The patient's main indication for LTBI treatment was recent arrival (within 5 years) from a high prevalence country. She presented to the emergency department with epigastric and right sided flank pain of one-week duration. The history goes back to one week prior to admission when the patient started to experience pain along with one episode of vomiting and dark (tea-colored) urine without any other urinary symptoms. She reported a severe pain of 8/10, relieved by sitting and lying forward and worsened by lying backwards. The patient denied any nausea, diarrhea, constipation, or fever prior to the presentation.

Her past medical history includes LTBI (6 months prior to the presentation with no laboratory and clinical monitoring for the isoniazid treatment) and dyslipidemia without any known liver disease. Her medications are Rimifon® (INH) 150 mg two tablets by mouth once daily and Panadol® (Acetaminophen) 500 mg two tablets by mouth as needed for headache. The patient was previously on Liponorm® (Atorvastatin) 10 mg one tablet by mouth once daily and Neurobion® (Vitamin B12, 200 mcg, Vitamin B6, 200 mg, and Vitamin B1, 100 mg) both of which were discontinued when INH was initiated (6 months prior to the presentation). The patient works as a housekeeper; she denied any known drug or food allergies, the use of recreational drugs, tobacco smoke, or alcohol.

Her vital signs were as follows: blood pressure of 120/78 mmHg, heart rate of 83 beats per minute, respiratory rate of 15 breaths per minute, and a body temperature of 36.2°C. Her review of systems was normal except for icteric sclera, pale conjunctiva, and epigastric tenderness. Pending laboratory results, she was given Perfalgan® (acetaminophen) 1 g IV STAT for pain and normal saline 0.9% 1 L every 24 hours. The patient's laboratory data upon admission are shown in Table 1.

The patient was admitted to the internal medicine service for further investigation. INH was discontinued and medication orders were the following: Profenid® (ketoprofen) 100 mg IV every 8 hours as needed for pain, Nexium® (Esomeprazole) 40 mg IV once daily, and Buscopan® (Scopolamine Butylbromide) 1 ampoule of 20 mg IV every 8 hours as needed for abdominal pain. Based on negative infectious serology, hepatitis A, hepatitis B, hepatitis C, cytomegalovirus (CMV), and Epstein-Barr virus were all ruled out. The patient had a positive CMV IgG Ab (160 AU/mL) suggesting a previous infection. Hepatic autoimmune disease was ruled out as well based on negative immunology markers. An abdominal ultrasound showed a normal liver size of 11 cm, perihepatic fluid effusion, and pericholecystic edema that are most probably related to hepatitis rather than cholecystitis. CT scan of the abdomen and pelvis showed a diffusely thickened gallbladder wall with neither the evidence of obstructive process nor portal vein thrombosis. After ruling out all the possible causes of acute hepatitis in our patient, the final diagnosis was made as INH-induced acute hepatitis.

Throughout the hospital stay, there was a slight reduction (around 15% from baseline) in liver function tests (LFTs); however INR and bilirubin remained elevated. The patient was clinically improving and her laboratory values were slightly better. Upon physician's order, the patient was discharged on Nexium 40 mg one tablet by mouth daily for one week. The patient did not resume the isoniazid treatment upon discharge after the initial hospital admission.

Six days later, the patient was readmitted to the hospital for abdominal distention and increased upper right quadrant pain that radiated to the flanks and increases with food intake and exertion. She also reported decreased appetite, fatigue, and dyspnea with exertion. Upon readmission, she presented with jaundice, icteric sclera, dark urine, and prominent ascites confirmed by wave fluid test. In the ER, she received Nexium 40 mg IV, Profenid 100 mg IV STAT, and NS 0.9% 500 mL every 24 hours which was increased to 1 L every 24 hours when her blood pressure dropped to 85/55 mmHg. Laboratory values were the following: total bilirubin 27.8 mg/dL, *γ*-GTP 95 U/L, ALP 283 U/L, ALT (SGPT) 873 U/L, AST (SGOT) 1582 U/L, amylase 164 U/L, lipase 65 U/L, Cr 0.66 mg/dL, and INR 2.28. Bilirubin was also detected in urine. Accordingly, the patient was admitted to the internal medicine floor for fulminant hepatic failure due to INH.

In an attempt to normalize INR, multiple administrations of Konakion® (Phytomenadione) were given. Profenid was discontinued due to the increased risk of bleeding and Buscopan 20 mg IV every 8 hours as needed for abdominal pain was ordered. Furthermore, Primperan® (Metoclopramide) 10 mg IV STAT, one vial of Albumin (20%) IV STAT, and morphine sulfate 2 mg SC STAT twice for pain were given. The patient had several episodes of hypotension responsive to fluid resuscitation with NS. The ultrasound showed diffused and clear moderate ascites as well as marked edematous thickening of the gallbladder wall with clear content related to intra-abdominal fluid effusion. The patient developed hypoglycemia (32–37 mg/dL) and oxygen saturation dropped to 86%. Due to deterioration, reduced level of consciousness, and poor response to oxygen, she was intubated and transferred to the intensive care unit.

On physical exam she was unresponsive, her pupils were middilated, and nonreactive, her lungs had few scattered rhonchi, and she had abdominal distention and ascites. She had metabolic acidosis (pH 6.8) and multiple doses of sodium bicarbonate were administered. The patient was consistently hypoglycemic and hypotensive despite fluid resuscitation and vasopressor administration. Serum creatinine increased from 0.66 mg/dL to 2.34 mg/dL in two days confirming acute kidney injury. She was also suffering from severe coagulopathy and hepatic encephalopathy. Additionally, blood cultures revealed a sensitive* Escherichia coli* and sputum cultures showed *α*-*Streptococcus* and* Neisseria* species. The patient had cardiorespiratory failure necessitating cardiopulmonary resuscitation (CPR). Spontaneous circulation was achieved after 15 minutes of CPR. Unfortunately, this was followed by a second cardiac arrest and despite maximal hemodynamic support, the patient passed away.

## 3. Discussion

The American College of Gastroenterology (ACG) clinical guidelines recommend the use of a specific formula (*R* = ALT/ULN ÷ ALP/ULN) to differentiate between three different types of drug-induced liver injury (DILI): acute hepatocellular (*R* ≥ 5), mixed (*R* 2–5), or cholestatic (*R* ≤ 2) [32]. The most common presentation of DILI is acute hepatocellular injury or cholestatic injury [33]. The former is more life-threatening (may lead to coagulopathy and encephalopathy) and is characterized by severe elevation of LFTs but mild elevation of ALP [34–36]. In cholestatic disease, there is a marked elevation of ALP, minimal elevation of LFTs, and it is accompanied by jaundice and pruritus [33]. Finally, the mixed type is somewhere in-between where the elevation of LFTs and ALP are both intermediate, resembling atypical hepatitis and granulomatous hepatitis [36]. INH fits the acute hepatocellular injury profile that is similar to acute viral hepatitis with a moderate to long latency period of 30 to more than 90 days [32, 33]. The reported patient's *R* score was 10 confirming the acute hepatocellular injury as expected. Other reports show latency period of approximately one week to three months; in most cases, however, the INH-induced hepatotoxicity can occur up to one year later or more [14]. An acute DILI may proceed to a chronic DILI in 15–20% of the cases. A chronic DILI is confirmed if the condition (clinical and laboratory) does not resolve 6 months after its onset. Usually, patients with cholestatic liver injury are at higher risk compared to those with acute liver injury [32].

We presented the case of a 65-year-old female with acute INH-induced hepatic failure that progressed dramatically in a short period of time leading to death. As mentioned earlier, hepatic involvement is a common side effect of INH. The patient's Naranjo adverse drug reaction probability score was 7, indicating a probable association [31]. Similarly, both the Yale Algorithm [37] and the WHO-UMC system for standardized case causality assessment [38] showed a probable association. It is also important to note that the patient received other potentially hepatotoxic medications: acetaminophen, by mouth and by intravenous injection, ketoprofen and esomeprazole, by intravenous injection and then by mouth, and metoclopramide. These medications could have contributed to the ultimate deterioration of the liver.

A recent case report presents a 53-year-old Japanese male receiving INH for LTBI [30]. Similar to our case, there was no routine laboratory monitoring. Our patient had a longer latency period (180 days versus 70 days) but a similar time from INH initiation until death (around 190 days). Both patients had a slight improvement in LFTs upon discontinuation of INH but remained extremely elevated. As for disease progression, hepatic encephalopathy and coagulopathy were noted in both cases. The Japanese patient developed sepsis and liver atrophy and died of liver failure, while our patient had acute kidney injury and metabolic acidosis and died of cardiorespiratory arrest. Liver transplant was an appropriate treatment option for both but was not feasible for different reasons.

The Center for Disease Control and Prevention published a report that quantified the frequency of severe adverse effects in patient receiving isoniazid for LTBI treatment during 2004–2008 [39]. Severe liver injury due to isoniazid was reported in 17 patients, 5 of whom underwent liver transplantation. Five patients died, including one patient who had a liver transplantation. The report emphasized the need for monthly clinical monitoring and counselling of patients receiving isoniazid for LTBI, including those with no evident additive risk factors for liver injury, to identify any treatment-associated adverse event.

According to The Diagnosis and Management of Idiosyncratic Drug-Induced Liver Injury published by the ACG, it is strongly discouraged to rechallenge a patient with a drug that was considered to be a causative agent for hepatotoxicity especially when the injury is severe (LFTs > 5x ULN). However, exceptions do occur in life-threatening situations when no other alternative is available [32]. As for the appropriate management, the first step would be withdrawing the offending agent and, if possible, other hepatotoxic agents as well [32]. Corticosteroids and ursodeoxycholic acid have been proposed as a treatment for DILI associated with acute liver failure (ALF) [40]; however, no robust evidence supports these treatment modalities [32]. The use of intravenous N-acetylcysteine (NAC) in non-acetaminophen acute liver failure (DILI in one subgroup) was tested in a prospective, double blind trial. Intravenous NAC did not enhance overall survival; however, it improved transplant-free survival in patients with early coma grade. Patients with advanced coma grades do not benefit from NAC and typically require emergency liver transplantation [41]. Another prospective double blind trial was conducted to determine the association between clinical benefit seen with IV NAC and improvement in hepatic function. In early grade coma patients with non-acetaminophen acute liver failure, the reduction in transplantation or death or of transplantation alone was associated with an improvement in ALT and bilirubin (parameters reflecting hepatocyte necrosis and bile excretion) but not in INR, creatinine, or AST [42]. Findings suggest an accelerated hepatic recovery with the use of IV NAC in such patients [42]. However, NAC use in pediatric non-acetaminophen related ALF showed conflicting results [43, 44].

A 4-month regimen of rifampin can be considered for persons who cannot tolerate INH, who have been exposed to INH-resistant tuberculosis, or who require a shortened duration of therapy for better completion rates [45]. It should not be used to treat HIV-infected persons taking some combinations of antiretroviral therapy [45]. In a pooled data from 3586 patients, a 4-month rifampin regimen was associated with a significant reduction in the risk of hepatotoxicity as compared to 9-month isoniazid therapy [46].

## 4. Conclusion

We reported a case of acute, fulminant, and fatal hepatotoxicity due to INH in a 65-year-old female with LTBI. Although hepatotoxicity is a well-known side effect of INH, mortality rates remain low as reported in the literature. It is important for clinicians and pharmacists to appropriately follow up patients, counsel them on the signs and symptoms of hepatotoxicity, and encourage them to report it.

## Figures and Tables

**Table 1:** Laboratory data upon admission.

Laboratory test	Result	Normal range
*Biochemistry*		
Total bilirubin	^*∗*^11.4 mg/dL	<1 mg/dL
Direct bilirubin	^*∗*^9.9 mg/dL	0–0.2 mg/dL
Indirect bilirubin	^*∗*^1.5 mg/dL	0-1 mg/dL
*γ*-GTP	^*∗*^202 U/L	<40 U/L
ALP	^*∗*^316 U/L	35–105 U/L
AST (SGOT)	^*∗*^2099 U/L	<33 U/L
ALT (SGPT)	^*∗*^1096 U/L	<34 U/L
CRP	^*∗*^1.54 mg/dL	<0.5 mg/dL
Amylase	^*∗*^115 U/L	28–100 U/L
Lipase	^*∗*^97 U/L	13–60 U/L
Total proteins	^*∗*^5.2 g/dL	6.6–8.7 g/dL
Albumin	^*∗*^2.9 g/dL	3.5–5.2 g/dL

*Hematology*		
WBC	^*∗*^4.8 × 10^3^/*µ*L	5.2–12.4 × 10^3^/*µ*L
RBC	4.5 × 10^6^/*µ*L	4.2–5.4 × 10^6^/*µ*L
Hb	13.7 g/dL	12–16 g/dL
PLT	237 × 10^3^/*µ*L	130–400 × 10^3^/*µ*L

*Coagulation*		
INR	^*∗*^1.58	1–1.3

^*∗*^Abnormal value.

*γ*-GTP: Gamma-Glutamyl Transpeptidase; ALP: Alkaline Phosphatase; AST: Aspartate Aminotransferase; SGOT: Serum Glutamic Oxaloacetic Transaminase; ALT: Alanine Aminotransferase; SGPT: Serum Glutamic Pyruvic Transaminase; CRP: C-Reactive Protein; WBC: White Blood Cell Count; RBC: Red Blood Cell; Hb: Hemoglobin; PLT: Platelet; INR: International Normalized Ratio.
